# Recent progress of hydrogel-based local drug delivery systems for postoperative radiotherapy

**DOI:** 10.3389/fonc.2023.1027254

**Published:** 2023-02-13

**Authors:** Yandong Xie, Mingxi Liu, Chang Cai, Chengkun Ye, Tangjun Guo, Kun Yang, Hong Xiao, Xianglong Tang, Hongyi Liu

**Affiliations:** ^1^ Department of Neurosurgery, Affiliated Nanjing Brain Hospital, Nanjing Medical University, Nanjing, China; ^2^ Department of Neurosurgery, The Suqian Clinical College of Xuzhou Medical University, Suqian, China; ^3^ Division of Life Sciences and Medicine, University of Science and Technology of China, Hefei, China; ^4^ Department of Neurosurgery, Affiliated Hospital of Xuzhou Medical University, Xuzhou, China; ^5^ Department of Neuro-Psychiatric Institute, Affiliated Nanjing Brain Hospital, Nanjing Medical University, Nanjing, China

**Keywords:** hydrogel, postoperative radiotherapy, radiation sensitization, local drug delivery, tumor recurrence

## Abstract

Surgical resection and postoperative radiotherapy remained the most common therapeutic modalities for malignant tumors. However, tumor recurrence after receiving such combination is difficult to be avoided because of high invasiveness and radiation resistance of cancer cells during long-term therapy. Hydrogels, as novel local drug delivery systems, presented excellent biocompatibility, high drug loading capacity and sustained drug release property. Compared with conventional drug formulations, hydrogels are able to be administered intraoperatively and directly release the entrapped therapeutic agents to the unresectable tumor sites. Therefore, hydrogel-based local drug delivery systems have their unique advantages especially in sensitizing postoperative radiotherapy. In this context, classification and biological properties of hydrogels were firstly introduced. Then, recent progress and application of hydrogels for postoperative radiotherapy were summarized. Finally, the prospects and challenges of hydrogels in postoperative radiotherapy were discussed.

## 1 Introduction

Malignant tumors, as the most complicated and challenging diseases, has received considerable attention all over the world (1). By now, the combination treatment of surgical resection and postoperative radiotherapy is still one of the most effective strategy, and is widely used for many kinds of tumors (2, 3). However, malignant tumor cells grow infiltratively and interweave with surrounding healthy tissues, which make it difficult to be completely resected. More importantly, the therapeutic effect of postoperative radiotherapy is limited by the radiation resistance of residual tumor cells (4, 5). These above reasons make the tumor easy to relapse, and the curative effect often dissatisfies us.

Numerous attempts (such as high-dose radiation, utilization of radiosensitizers, chemoradiotherapy and immunoradiotherapy) have been made to address the pitfalls of surgery and postoperative radiotherapy (6–9). Unfortunately, it has been proved that high-dose radiation can cause irreversible damages to the peritumoral normal tissues and further cause various adverse side effect in patients (10). Traditional radiosensitizers or chemotherapeutics could not effectively penetrate into tumor tissues, which limits their radiosensitization effects after systemic administration. In addition, the systemic toxicity, gastrointestinal side effects, myelosuppression and some other side effects can also be caused by such systemic administration (11, 12). Immunoradiotherapy has attracted great research interests in recent years. Tumor-associated antigens (TAAs) can be released from radiation-induced apoptotic cancer cells, which further cause antitumor immune responses (13). But immune-related adverse events (irAEs), which produced by an overactive immune response against healthy organs, can seriously affect the therapeutic efficacy of immunoradiotherapy (14, 15). The nonspecific distribution of conventional antitumor drugs inhibits the effectiveness of postoperative radiotherapy, and the repeated systemic administration make antitumor therapy more complicated. As a result, local drug delivery systems, especially hydrogels, have been explored to address these constraints, with the potential to simultaneously improve antitumor efficacy and minimize systemic side effects.

**Figure 1:**
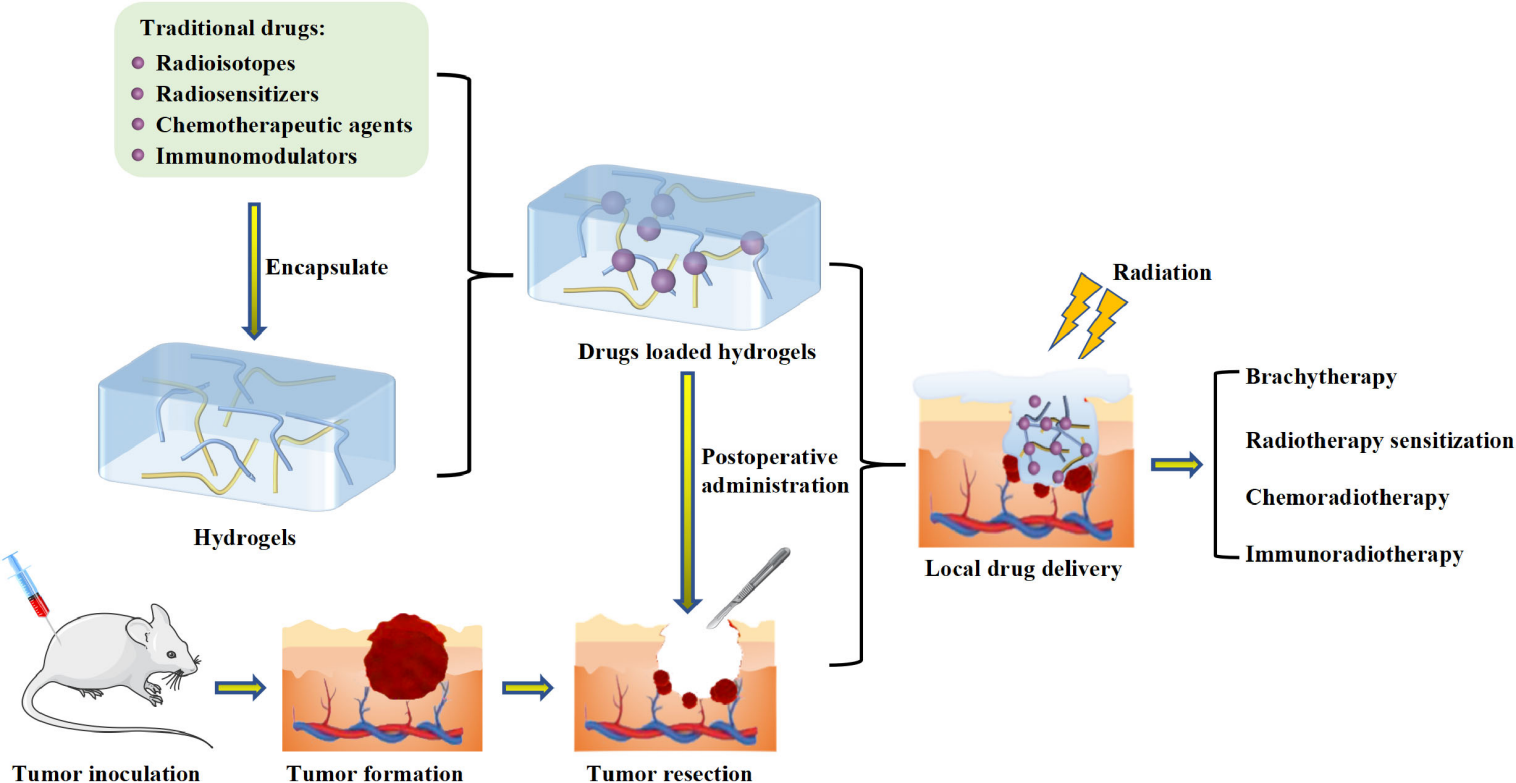
Schematic illumination of the application of hydrogel-based local drug delivery systems for postoperative radiotherapy.

With the fast development of material engineering and molecular biology, hydrogel has opened up new avenues for the treatment of cancer. Hydrogel refers to a three-dimensional network gel constituted with hydrophilic polymers, which has good permeability and excellent biocompatibility. It can be directly applied to the pathological tissue and has a wide range of applications prospects in the field of biological medicine (16–22). Hydrogel can be injected or sprayed into postoperative cavities, *in situ* gel and circumvent the biological barrier, such as blood brain barrier (23). Before reaching the tumor cells, the agents can be protected by hydrogel from being affected by severe surroundings. And the plasma half-life can be prolonged (24). To optimize therapy effectiveness and minimize systemic drug distribution, hydrogel can be designed to delivery drug to specific cells or tissues (25). In addition, the unique porous structure of the hydrogel can optimize drug sustained release properties. The controlled release properties can also be obtained by utilizing an elaborated stimuli-responsive hydrogel delivery system (26). The strategy of coadministration of different pharmaceuticals can similarly be achieved by hydrogel delivery systems, which can overcome drug resistance and boost therapeutic efficacy (27). From the above information, we can see that the hydrogel-based drug delivery systems have their unique advantages in postoperative radiotherapy. Traditional drugs formulations (such as radioisotopes, radiosensitizers, chemotherapeutic agents or immunomodulators) were recorded by encapsulating into hydrogels and combing with postoperative radiotherapy to inhibit tumor recurrence (**Figure 1**). This local drug delivery method can avoid the nonspecific distribution of traditional drugs, sensitize radiotherapy and achieve the combination of multiple treatment modalities.

In this review, we first introduced the classification and biological characteristics of hydrogels. Next, the applications of hydrogel-based local drug delivery systems for postoperative radiotherapy were further systematically investigated. Lastly, the prospects and challenges of hydrogel-based local drug delivery systems in postoperative radiotherapy were discussed.

## 2 The classification and biocompatibility of hydrogels

### 2.1 Classifications of hydrogels

According to different standards, hydrogels are divided into different classifications (**Table 1**).

a) Based on the original materials, it can be divided into natural hydrogels and synthetic hydrogels (28). The former includes alginates hydrogel, chitosan hydrogel, collagen hydrogel, hyaluronic acid hydrogel, etc (35–37). The latter is synthetized by a variety of polymers, including polyethylene glycol (PEG), poly glycolic acid (PGA) and poly lactic-co-glycolic acid (PLGA) (38–40).
b) According to the cross-linking junction types, hydrogels can be divided into physical cross-linked hydrogels and chemical cross-linked hydrogels. On the molecular level, noncovalent bonding interactions lead to physically cross-linked hydrogels. Although the connections are typically fleeting, they are enough to make hydrogels insoluble in aqueous media. Irreversible covalent cross-linking interaction produces chemically cross-linked hydrogels. Linear or branching polymers directly come into contact with one another by chemical cross-linking reaction, which leads to extremely high mechanical strength (29, 30).
c) In terms of network charge, three groups of hydrogels are categorized: anionic, cationic and neutral hydrogels. The whole network’s charge is determined by the polymer’s charge (31, 32).
d) In accordance with the polymeric compositions, hydrogels are divided into three classes: (I) Homopolymeric hydrogels (composed of a single monomer species). (II) Copolymeric hydrogels (obtained from two or more monomer species, at least one being hydrophilic). (III) Multipolymeric hydrogels (made up of two independent and cross-linked chains of polymers) (33).
e) Hydrogels can also be generally divided into conventional and intelligent hydrogels. Conventional hydrogels refer to hydrogels that are not sensitive to environment, such as temperature or pH, etc. Conventional hydrogels have a few fundamental properties. The unique porous architectures of conventional hydrogels can facilitate molecular penetration. The hard chain architecture can resist dissolution, and the flexible architecture can make conventional hydrogels extendable and collapsible (34). Intelligent hydrogels, also named as stimuli-responsive hydrogels, have the capacity to alter self-physical characteristics (swelling ability, mechanical properties, molecular diffusion, etc). It can response to a variety of environmental stimuli, such as temperature, light, pH, enzyme, electric fields, and some other biological factors (41–45). In the tumor microenvironment, stimuli-responsive hydrogels can modify its own rheological behavior (46). By encapsulating pharmaceuticals in unique response hydrogel, the medication release could be better controlled. For example, pH-responsive hydrogels can control the drug release in the acidic tumor microenvironment (47).

**Table 1:** The classification of hydrogels based on different standards.

Classification standards	Kinds of hydrogels	References
Material	Natural hydrogels Synthetic hydrogels	(28)
Crosslinking manner	Physically hydrogels Chemically hydrogels	(29, 30)
Electrical charge	Anionic hydrogels Cationic hydrogels Neutral hydrogels	(31, 32)
Polymeric composition	Homopolymer hydrogels Copolymer hydrogels Multipolymer hydrogels	(33)
Type of stimuli responsive	Conventional hydrogels Intelligent hydrogels	(34)

### 2.2 Biocompatibility of hydrogels

Biocompatibility is the biological property that a material can tolerate the action of various systems of the host, while the material can still remain relatively stable and not be rejected or destroyed. Excellent biocompatibility is a prerequisite for hydrogels to be widely used in biomedical fields (48). Polysaccharide is a well-known raw material of hydrogel. Zhao et al. detected its cytotoxicity *in vivo* and found that it did not produce acute toxic reaction to the blood system (49). Zhang et al. investigated the cytotoxicity of fibrin hydrogel to DC 2.4 cells and C57BL/6 mice. The *in vitro* data showed that the cell survival rate was more than 90% after coincubation with fibrin hydrogel for 24 h, and the *in vivo* H&E staining assay did not show obvious inflammatory response (50). Liu et al. evaluated the cytotoxicity of polymer HA-DEG/UPy hydrogel on 3T3 fibroblasts. The experimental results showed that HA-DEG/UPy hydrogel had almost no toxicity to 3T3 fibroblasts cells and the cell viability remained almost 100% (51). The high biocompatibility of hydrogel effectively avoids the generation of systemic toxicity, it can be safely applied in the treatment of diseases.

## 3 The application of hydrogel-based local drug delivery systems in postoperative radiotherapy

Hydrogels have the capacity to encapsulate various drugs through blending them together in precursor solution, which are then converted into three-dimensional mesh structure gel. After *in vivo* administration, the therapeutic substances encapsulated in hydrogels localized in the target area and the drug delivery systems sustained the drug release for an extended time (52, 53). Hydrogel based local drug delivery systems have been explored to encapsulate traditional drug (such as radioisotopes, radiosensitizers, chemotherapeutic agents, and immunotherapy agents), which can enhance the therapeutic efficacy of postoperative radiotherapy.

### 3.1 Hydrogels for the local delivery of radioisotopes in postoperative radiotherapy

Brachytherapy, also known as internal radiotherapy, is a kind of radiotherapy that deliver sealed radiation source to tumor tissue or post-operative tumor cavity. This therapeutic modality has been applied to the treatment of a variety of tumors (54, 55). If administered systemically, the non-specific distribution of radioisotopes have harmful effects on the normal organs of patients. Hydrogel drug delivery systems can directly transfer radioisotopes to the tumor area. It can avoid the non-specific distribution of radioisotopes and have received great attention. Meng et al. designed a CuS/^131^I-PEGDA/AIPH hydrogel, in which copper sulfide responsed to the near-infrared laser to raise the temperature of the tumor-bearing region. When the AIPH thermal initiator was activated, the polymer matrix PEGDA began to gel and ^131^I (Iodine-131) was effectively preserved in the tumor cavity. The *in-situ* gelation of PEGDA triggered by NIR could prolong the retention time of ^131^I in tumor cavity and prevent it from leaking into adjacent normal tissues. What’s more, hyperthermia, induced by NIR responsive photothermal hydrogels, could boost blood circulation and further alleviated the tumor hypoxia microenvironment. As a result, the radiation sensitivity of tumor cells could be improved (56).

**Figure 2:**
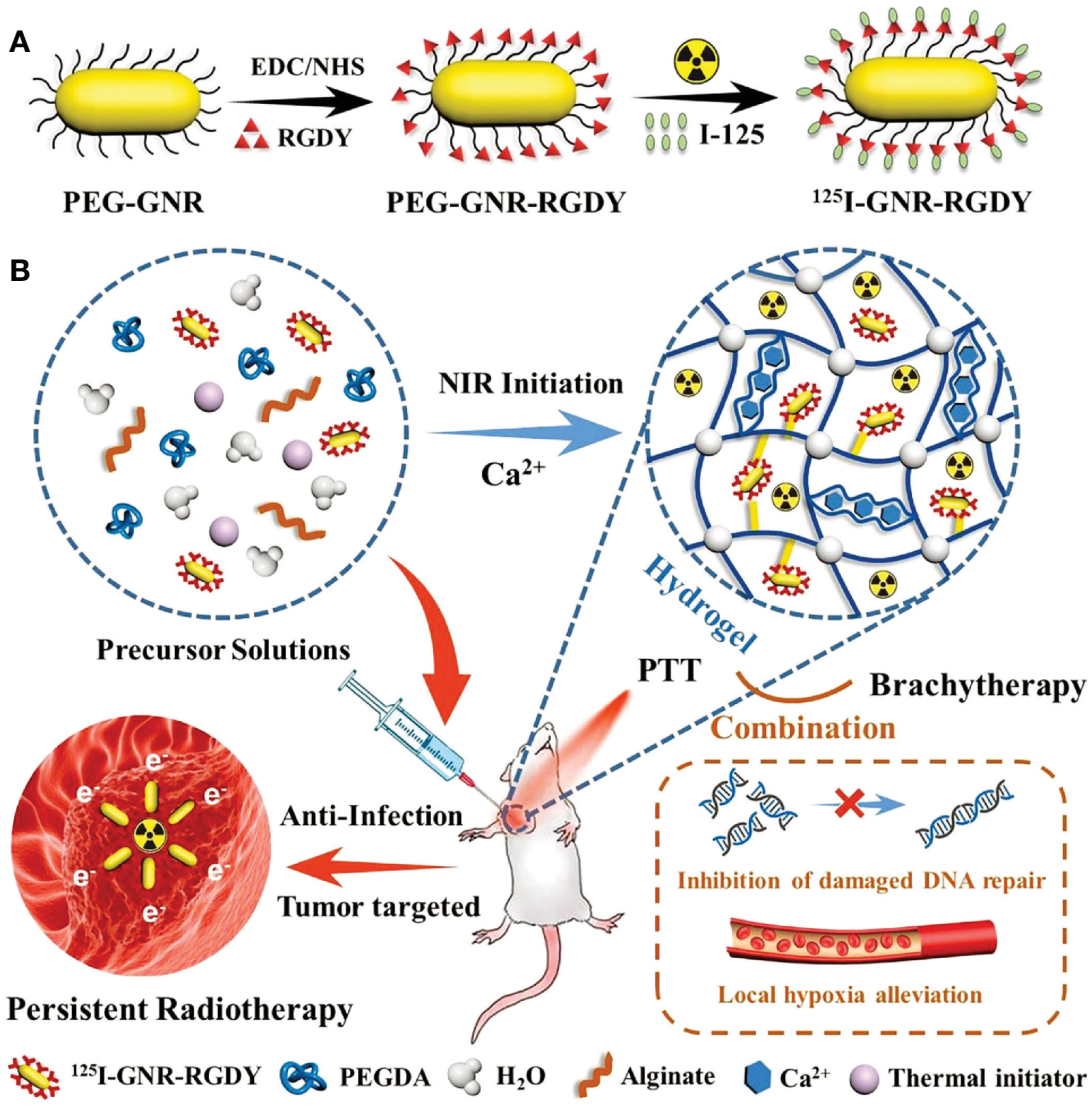
**(A)** ^125^I-GNR-RGDY and **(B)** nanocomposite double-network GPA hydrogel and their theranostic application for inhibition of postoperative breast cancer recurrence and wound infection through synergistic brachytherapy and photothermal therapy (22).

Apart from ^131^I, the hydrogel-based delivery systems could also deliver some other radioisotopes, such as ^125^I (Iodine-125) and ^188^Re (Rhenium-188). ^125^I implantation had been demonstrated to be an effective approach to eradicate tumor cells, and it only cause minor damage to adjacent normal cells (57). Wu et al. constructed an injectable near-infrared induced polymerized hydrogel (^125^I-GPA), which could continuously release ^125^I-GNR-RGDY into tumor tissues. It had been successfully applied to the surgical resection model of breast cancer (22). In this experiment, hyperthermia induced by near-infrared radiation could eradicate potentially pathogenic bacteria and was helpful to prevent postoperative wound infection. Besides, the RGD peptide modification on the surface of ^125^I-GNR-RGDY could target tumor cells and achieve more accurate brachytherapy (**Figure 2**). The recurrence time of different treatment groups demonstrated that the combination treatment of ^125^I-GPA + NIR suppress tumor growth more effectively than GAP + NIR. And no tumor recurrence was observed in ^125^I-GPA + NIR treated group.

**Figure 3:**
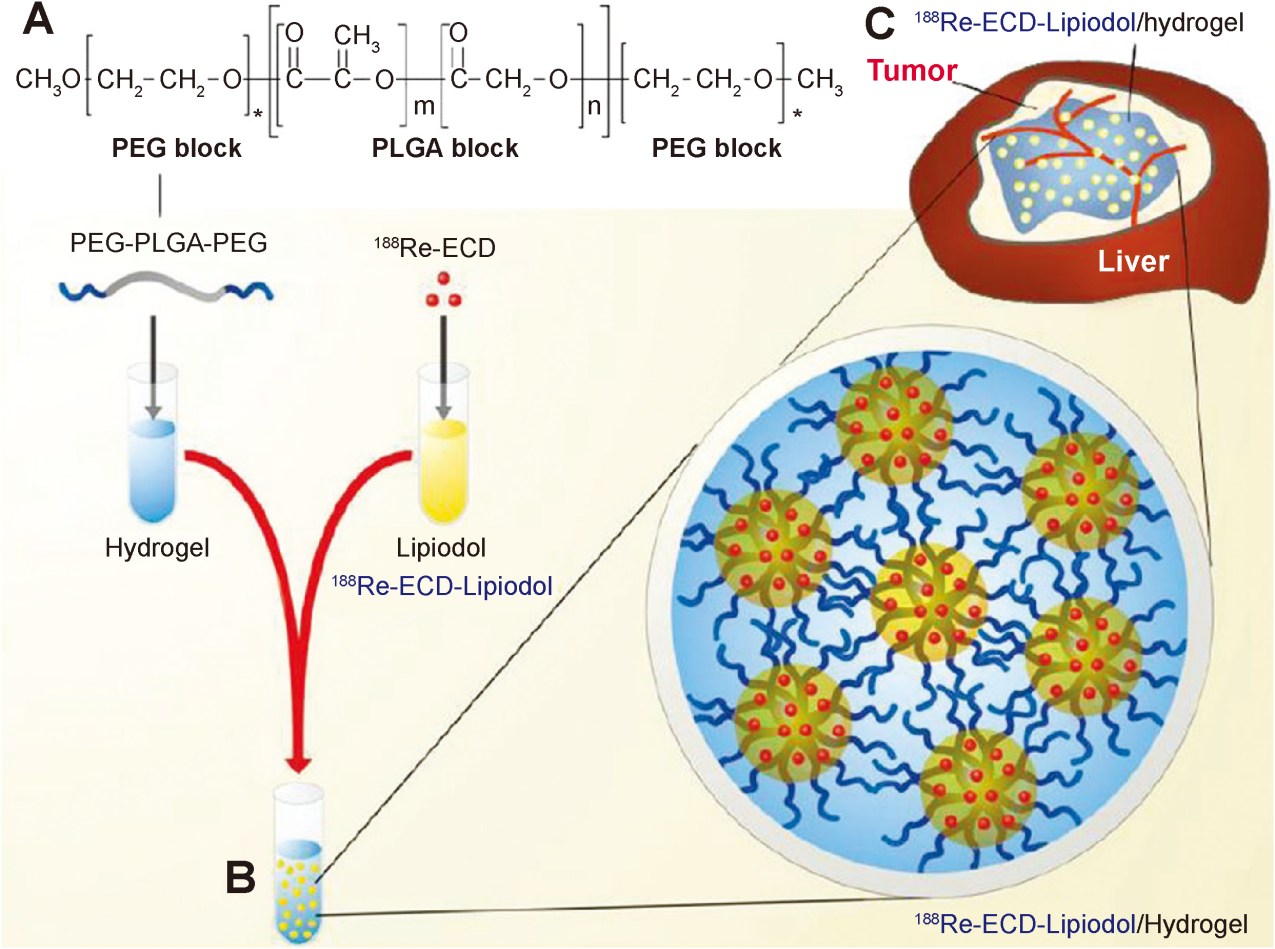
PEG-PLGA-PEG chemical structure, preparation of ^188^Re-ECD-Lipiodol/hydrogel, and hepatoma animal model treatment. **(A)** Chemical structure of PEG-PLGA-PEG. **(B)** Optical images of ^188^Re-ECD-Lipiodol/hydrogel, prepared by mixing PEG-PLGA-PEG (30% w/w) and ^188^Re-ECDLipiodol in a 1:1 ratio. **(C)** ^188^Re-ECD-Lipiodol/hydrogel was administered to N1-S1 hepatoma-bearing rats *via* intratumoral injection (60).

^188^Re is an another radionuclide with β-ray that has been frequently applied in radioisotope therapy (58, 59). Shi et al. encapsulated the radiopharmaceutical ^188^Re-EL in a temperature-sensitive hydrogel to construct a hybrid radioactive thermosensitive hydrogel system (^188^Re-EL/hydrogel), which synergistically potentiated the therapeutic efficacy of ^188^Re-EL on hepatocellular carcinoma (**Figure 3**) (60).

Overall, encapsulating radionuclides into hydrogels exhibit obvious advantages in treating tumors. Compared with conventional radioisotope implantation, hydrogel can prolong the retention time of radionuclides in tumors, which is conducive to improving the effectiveness of internal radiotherapy. At the same time, hydrogel can effectively prevent the implanted radioisotopes from migrating to adjacent normal cells and reduce the adverse reactions of radionuclides.

### 3.2 Hydrogels for the local delivery of radiosensitizers in postoperative radiotherapy

Ionizing radiation, which includes high-energy X-rays, gamma rays, heavy ions, as well as electrons, is used in radiotherapy to directly produce DNA damage, or indirectly induce cell death by stimulating the generation of massive numbers of toxic reactive oxygen species (ROS) (61). However, abnormal vasculature and insufficient blood flow together contribute to hypoxia in solid tumors, which significantly reduces the effectiveness of radiotherapy and leads to radiation resistance (62). Besides, radiotherapy has the characteristic that significantly suppresses tumor growth in a dose-dependent manner, and its effectiveness is typically restricted by the maximum radiation dose which can be applied to the tumor area without causing serious damage to surrounding tissues (63). Therefore, radiosensitizers (such as nitroimidazoles, cisplatin, metal-based nanoparticles and so on) have been developed to minimize peripheral tissue lesion while maintaining sufficient ionization damage to tumors (64, 65). Hydrogels, as local drug carriers, can be injected into surgical cavities and overcome the limitation of physiological barriers for traditional drug delivery. It has been applied to the administration of various radiosensitizers to tumor areas, generating a synergistic radiosensitive effect (66).

**Figure 4:**
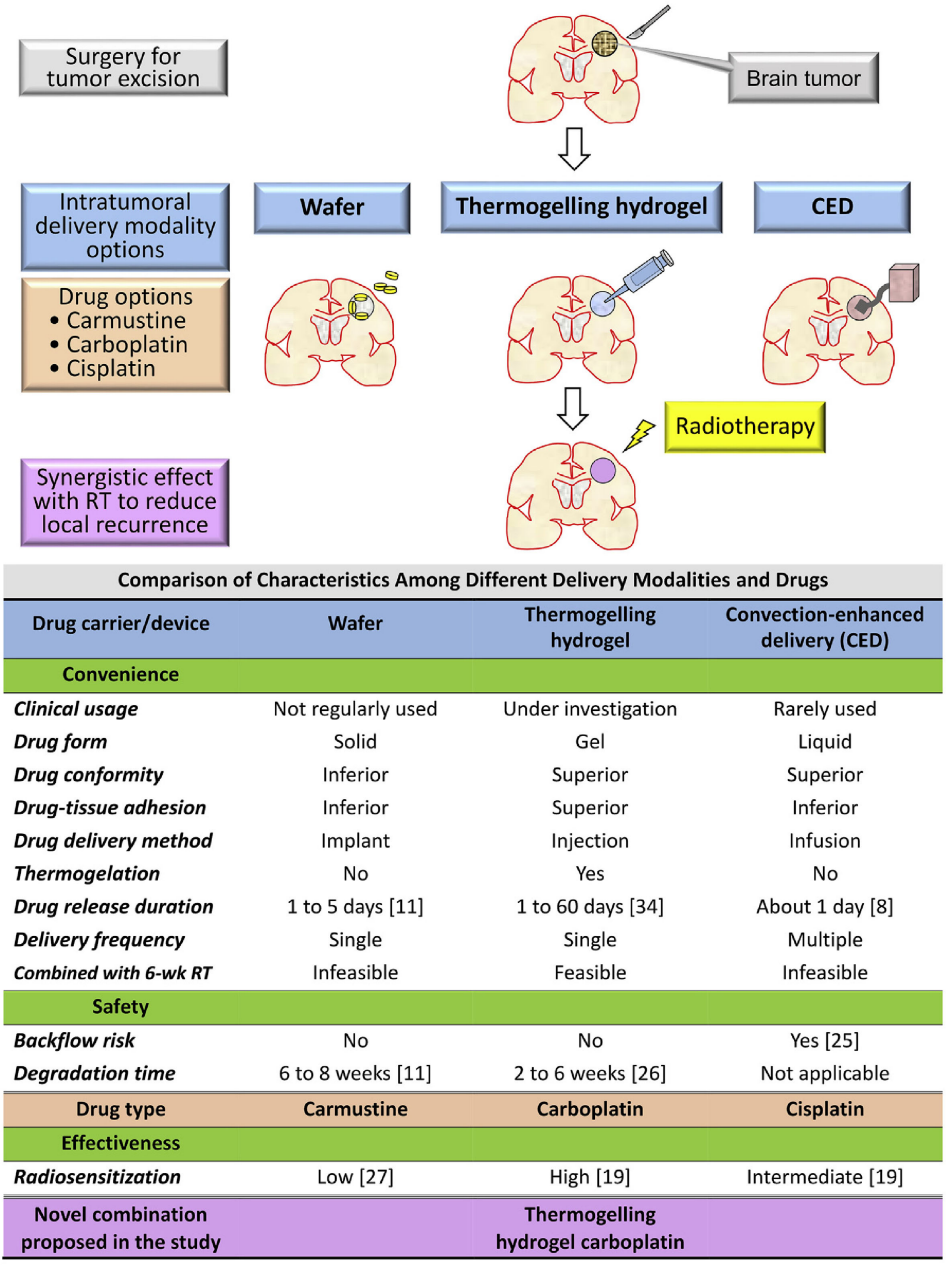
Rationale and purpose of this study: To compare the characteristics of intratumoral delivery modalities and drugs for malignant gliomas and propose a novel combination to satisfy the unmet clinical need of convenience, effectiveness, and safety. RT, radiotherapy; wk, week (70).

Glioblastoma (GBM), the most common primary malignant brain tumors, have a median survival of only 12-15 months (67). The radiation tolerance of glioma cells limits the therapeutic effect of radiotherapy. Worse still, the presence of blood brain barrier (BBB) makes traditional anticancer agents ineffective, resulting in unexpected GBM recurrence and poor prognosis for patients (68, 69). To overcome this limitation, Liang et al. reported a thermal-sensitive hydrogel encapsulating carboplatin that can be injected into the surgical cavity, circumventing BBB and delivering radiosensitizer to improve the postoperative radiotherapy of GBM (**Figure 4**) (70). In this research, carboplatin was used as radiosensitizer to prevent radiation-induced DNA damage from being repaired, making radiation therapy more sensitive. This research found that intratumorally administered hydrogels have the advantages of lowering systemic toxicity, bypassing the BBB and simplifying the drug delivery frequency. It is indicated that the injection of hydrogel systems containing smart radiosensitizers into the surgical cavity, combined with ionizing radiation, is a promising treatment strategy for glioblastoma.

**Figure 5:**
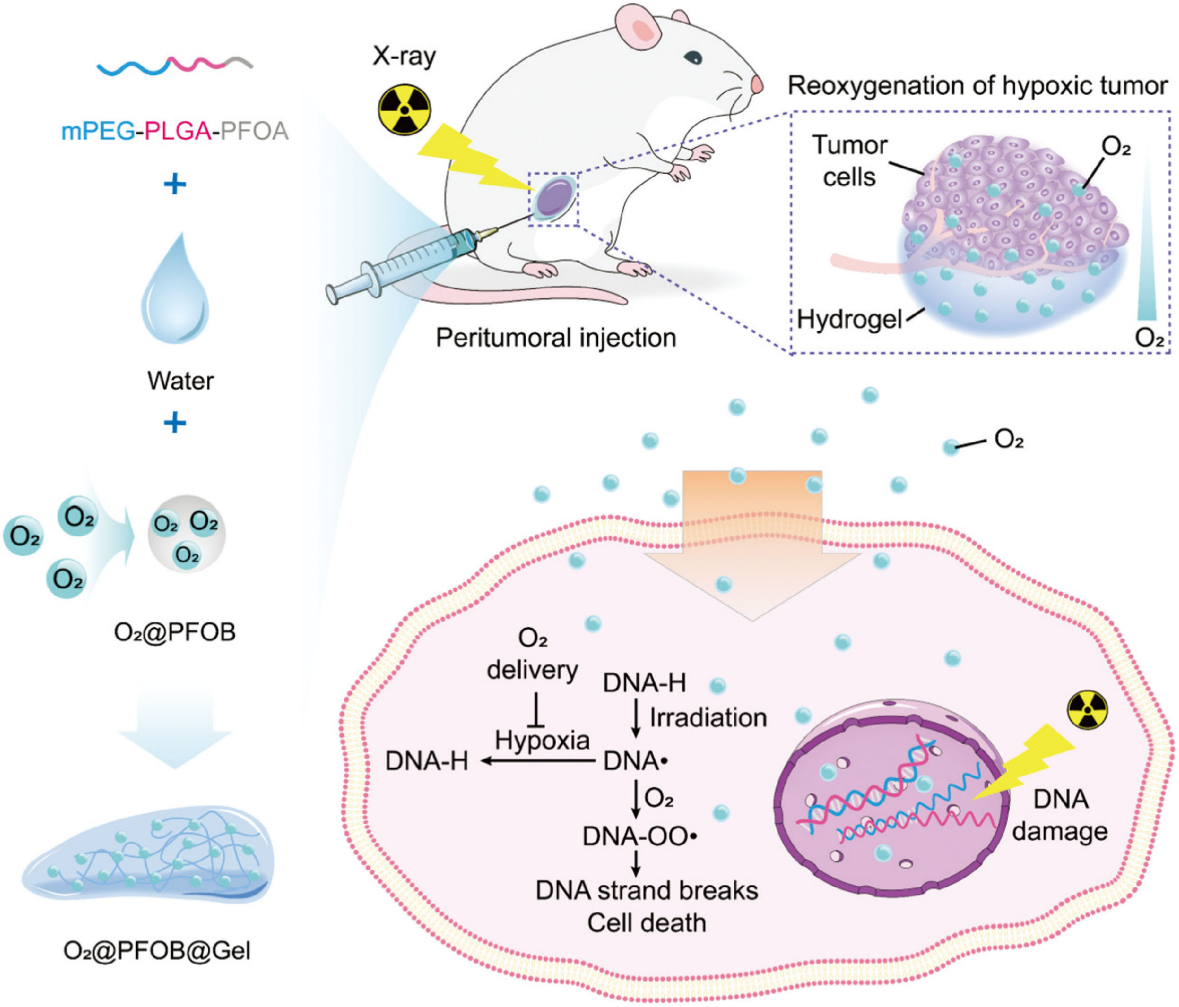
Schematic representation of the preparation of an oxygen-enriched thermosensitive composite hydrogel (O_2_@PFOB@Gel) and its radiosensitizing effect on tumor. The thermosensitive composite hydrogel undergoing a thermoreversible sol–gel transition is made up of a PFOA-modified mPEG–PLGA diblock copolymer and PFOB. After peritumoral injection of the O_2_@PFOB@Gel system, the hypoxic tumor microenvironment gains great alleviation *via* the sustained release of oxygen. As the released oxygen reacts with a damaged segment of DNA strand that is induced by X-ray exposure, the repair of DNA strand breaks is inhibited, resulting in more cell death. Consequently, a radiosensitizing effect is achieved (16).

Oxygen, acknowledged as the definitive hypoxic cell radiosensitizer, has been widely used to enhance radiotherapeutic efficacy (71, 72). Yang et al. constructed an oxygen-enriched thermosensitive hydrogel that steadily provided exogenous oxygen playing as a potent radiosensitization role in increasing the radiosensitivity of tumor cells (16). As shown in **Figure 5**, exogenous oxygen sustained released from O_2_@PFOB@Gel and induced reoxygenation of hypoxic tumor, successfully eliminating the hypoxia-related radiation resistance and significantly suppressing the tumor growth.

Sunitinib, as a small molecule multikinase inhibitor targeting VEGF receptors, has powerful antiangiogenic properties and has been proposed as a radiosensitier (73, 74). Fu et al. introduced a matrix metalloproteinase (MMP) -responsive hydrogel loaded with sunitinib nanoparticles (NS-MRH) to improve radiosensitivity and prevent local breast cancer recurrence (75). By being injected into the postoperative cavity and continuously releasing sunitinib, the constructed hydrogel system in this study successfully sensitized radiation and inhibited tumor recurrence.

### 3.3 Hydrogels for the local delivery of chemotherapeutic agents in postoperative radiotherapy

Postoperative concurrent chemoradiotherapy, which involves the simultaneous administration of chemotherapeutics and radiotherapy following surgery, has become the standard treatment for various solid tumors including lung cancers, esophageal cancer, gastrointestinal malignancies, as well as brain tumors. During the treatment of radiotherapy, normal tissues are also subjected to radiation exposure. When the radiation dose exceeds the maximum tolerance level of normal tissues, radioactive necrosis will occur (76). Traditional chemotherapeutic agents have been proven to sensitize radiotherapy in solid tumors, but their nonspecific tissue distribution usually causes severe damages to normal tissues and organs (77).

**Figure 6:**
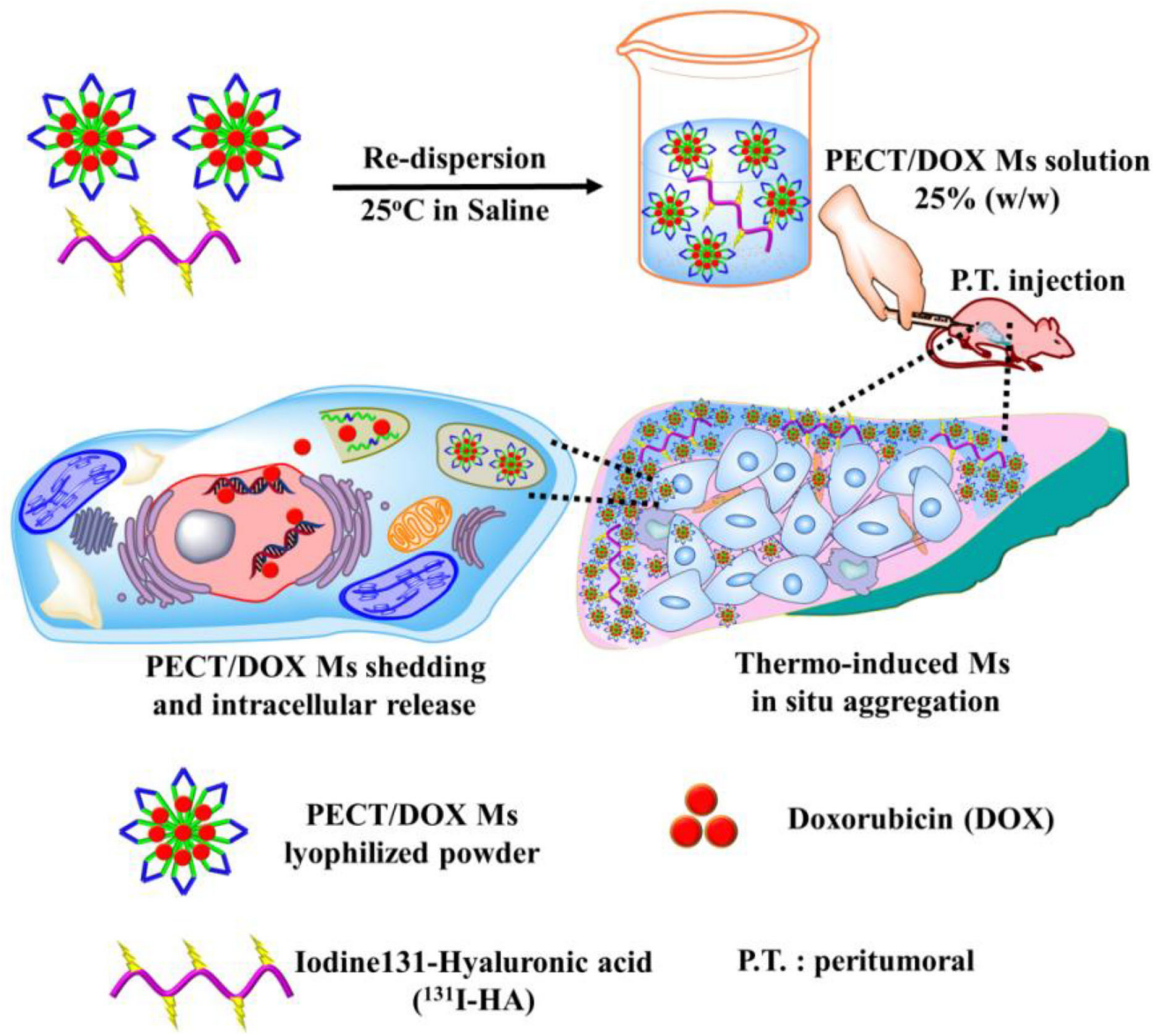
The schematic diagram of the formation of PECT/DOX MHg as the nanodrug and radionuclide reservoir and its following action model of *in situ* chemoradiotherapy (82).

Because of the unique characteristics of intraoperative administration, sustained drug release and high drug loading, hydrogel has been widely employed in postoperative chemoradiotherapy (78, 79). Doxorubicin (DOX), as a broad-spectrum anticancer drug, can effectively inhibit the synthesis of RNA and DNA to eliminate tumor cells. But the toxic side effects of doxorubicin, such as myelosuppression and myocardial toxicity, seriously limit its use in the clinic (80, 81). In order to minimize DOX toxicity, intelligence hydrogels have been employed. Huang et al. designed a thermos-responsive hydrogel (PEG-PLGA-PEG) for the co-delivery ^131^I as a radioactive source and DOX/PECT micelles as a chemotherapeutic to achieve combined chemoradiotherapy (**Figure 6**) (82). This hydrogel delivery system was confirmed that it enhanced the synergistic therapeutic efficacy of chemoradiotherapy and alleviated the toxic side effects of DOX. Another study utilizing DOX as the major sensitization ingredient was presented by Peng and his coworkers. ^188^Re-Tin and liposomal DOX were encapsulated in a thermoresponsive hydrogel (PCL-PEG-PCL) to maximize the ^188^Re therapeutic efficacy for hepatocellular carcinoma (83).

### 3.4 Hydrogels for the local delivery of immunomodulators in postoperative radiotherapy

It has been confirmed that radiation can enhance antitumor immune responses by inducing immunogenic cell death (ICD) (84–86). Danger associated molecular patterns (DAMPs) released by ICD promote the maturation of antigen-presenting cells (APCs), the infiltration of cytotoxic T lymphocytes (CTLs) and the release of associated cytokines (87, 88). CTLs could cooperate with cytokines such as interferon-γ(IFN-γ) and tumor necrosis factor-α (TNF-α) to reverse the immunosuppressive tumor microenvironment into an immunogenic phenotype, increasing immunotherapy responses of tumor cells (89, 90). However, the increased sensitivity of immunotherapy in post-radiation therapy is only to a certain extent and radiotherapy alone could not effectively suppress distant tumor growth (91). To enhance the immune efficacy of radiation and mitigate the systemic toxic side effects of immunotherapy drugs, hydrogels were used to deliver immunomodulators after injection into the tumor cavity and obtain combination effect with radiotherapy for various tumor treatment.

**Figure 7:**
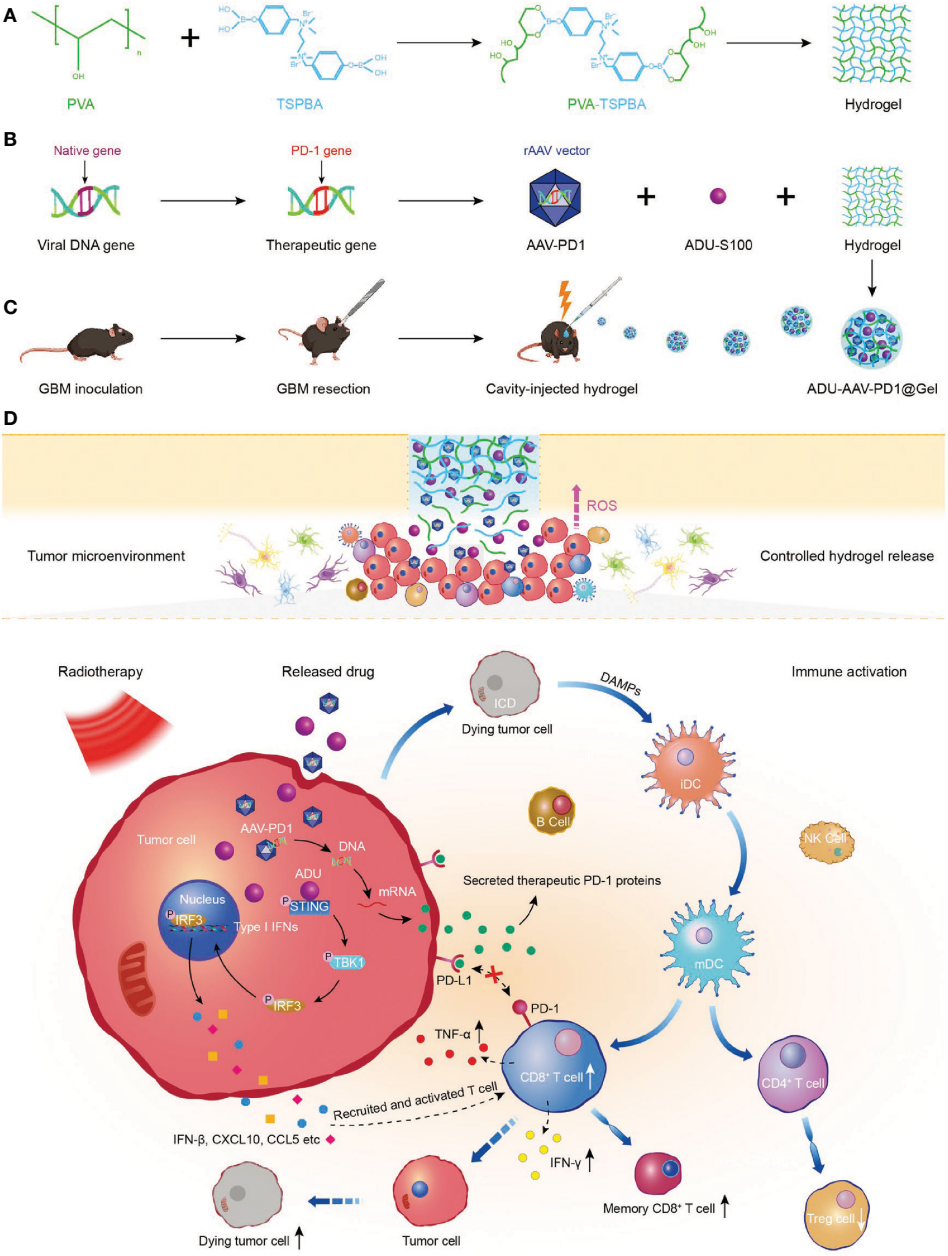
Preparation and mechanism of ADU-AAV-PD1@Gel. **(A)** Schematic illustration of the preparation of PVA-TSPBA hydrogel. **(B)** Flowchart of generation of the AAV-PD1 and formation of the ADU-AAV-PD1@Gel. **(C)** The ADU-AAV-PD1@Gel combined with RT treatment was performed in the GBM resection mice model. ADU-AAV-PD1@Gel was injected into the GBM resection cavity on day 11 after the GBM inoculation. The RT was performed on day 12, 13, and 14. **(D)** Mechanism of combining ADU-AAV-PD1@Gel with RT treatment for synergetic radioimmunotherapy of post-resection glioma (92).

Sun et al. developed the ADU-AAV-PD1@Gel, a ROS-responsive hydrogel for localized radioimmunotherapy in the glioblastoma resection mouse model (92). As shown in **Figure 7**, STING agonist (ADU) was used to improve tumor immunogenicity and adeno-associated virus-based-PD1 (AAV-PD1) was utilized to secret therapeutic PD-1 proteins to block the interaction between PD-1 and PD-L1 for restoring immunotherapy. ADU and AAV-PD1 were encapsulated together in a ROS-responsive hydrogel and orthotopically injected into the postoperative glioma cavity, enhanced the immune response induced by radiotherapy. This combination treatment effectively inhibited the recurrence of glioma after operation and prolonged the survival time of the model mice.

Immune adjuvants have been shown to be highly effective in enhancing antitumor immune responses and promoting radiation-induced ICD (93–95). CpG oligodeoxynucleotide (CpG ODN) is a toll-like receptor 9 (TLR9) agonist, which is widely used as an immune adjuvant (96, 97). Liu et al. created an ATP-responsive sodium alginate hydrogel containing ATP-specific aptamers and CpG ODNs for local immunoradiotherapy (98). CpG ODNs were released in response to ATP secreted from dying cells after radiotherapy, and the released CpG had been confirmed to have the ability to boost the antitumor immunotherapy of ionizing radiation at low doses. Notably, when combined with systemic administration of PD-L1 antibody, the constructed smart hydrogel exhibited excellent ability to eliminate established tumors and inhibit distant tumor metastasis, the latter of which was made possible by long-term immune memory effect.

Taken together, these findings demonstrated that hydrogel delivery of immunomodulators can be directly injected into the operation cavity, successfully bypassing physiological barriers (such as BBB) and reducing the off target effects of immunostimulatory agents, enhancing radiation-induced immune responses, and having significant efficacy in suppressing local and distant tumors.

## 4 Conclusions and prospects

*In situ* hydrogel drug delivery system has received considerable concerns and numerous successful applications of hydrogel as anti-cancer local delivery carrier has been witnessed in recent decades. Ideal hydrogels should present biodegradable property, avoiding producing harmful substance during degradation, has high drug loading efficiency and minimize the negative effects of anti-tumor drugs. In this context, we systematically summarized the recent researches of hydrogel-based drug delivery systems in postoperative radiotherapy. Different types of anti-tumor drug agents, such as radioisotopes, radiosensitizers, chemotherapeutic molecules or immunomodulators can be encapsulated in hydrogels to achieve combined therapeutic effect for postoperative radiotherapy, which has been proven to effectively sensitize radiation therapy and successfully suppress tumor growth and recurrence.

Although many advances have been achieved in the application of hydrogels for postoperative radiotherapy, there are still some challenges to be considered. From the perspective of clinical translation, hydrogels still could not completely replace the existing adjuvant treatment methods. The design of existent hydrogel drug delivery system is too complex, which is a great challenge to the quality control of products. Hydrogels showed controlled local drug release, but it was difficult to accurately control the drug release behavior per unit time. Therefore, simplifying the preparation procedure of hydrogels, improving the accuracy of drug release and release rate will hopefully further improve the application prospect of hydrogels in postoperative radiotherapy.

## Author contributions

YX and HL designed the manuscript. YX and CY were responsible for the writing of the abstract and introduction of the manuscript. ML and TG were responsible for the writing of the second part of the manuscript. CC and KY were responsible for the writing of the third part of the manuscript. YX and XT were responsible for writing the fourth part of the manuscript. HX, XT and HL revised the manuscript and gave final approval of the version to be published. All authors contributed to the article and approved the submitted version.
